# Growth patterns of the pan‐European freshwater mussel, *Anodonta anatina* (Linnaeus, 1758) (Bivalvia: Unionidae), vary with sex and mortality in populations

**DOI:** 10.1002/ece3.7250

**Published:** 2021-02-09

**Authors:** Tomasz Müller, Anna Maria Labecka, Katarzyna Zając, Marcin Czarnoleski

**Affiliations:** ^1^ Faculty of Biology Institute of Environmental Sciences Jagiellonian University Kraków Poland; ^2^ Institute of Nature Conservation Polish Academy of Sciences Kraków Poland

**Keywords:** energy allocation, glochidia brooding, growth curves, indeterminate growth, life history

## Abstract

1. Post‐maturation growth leading to indeterminate growth patterns is widespread in nature. However, its adaptive value is unclear. Life history theory suggests this allocation strategy may be favored by temporal pulses in the intensity of mortality and/or the capacity to produce new tissues.
2. Addressing the origin of indeterminate growth and the variability of growth patterns, we studied the growth of duck mussels, *Anodonta anatina*, a pan‐European unionid, in 18 Polish lakes. For each population, the sex, size, and age of collected mussels were measured to estimate Bertalanffy's growth curve parameters. We integrated information on *A. anatina* mortality rates, lake trophy, biofouling by zebra mussels, *Dreissena polymorpha*, and the prevalence of parasitic trematode larvae to identify selective conditions in lakes.
3. We found two sources of mortality in *A. anatina* populations, pertaining to adverse effects of zebra mussel biofouling and trophy state on mussel survival. Additionally, populations with heavier biofouling presented a smaller abundance of parasites, indicative of a relationship between filtering intensity and contraction of water‐borne trematode larvae by filtering *A. anatina*.
4. Consistently for each sex, populations with a greater trophy‐related mortality were characterized in *A. anatina* by a smaller asymptotic size *L_max_*, indicative of a life history response to mortality risk involving early maturation at a smaller body size. In all populations, females featured higher mortality and larger asymptotic size versus males.
5. Our findings support a theoretical view that adaptive responses to selection involve adjustments in the lifetime resource allocation patterns. These adjustments should be considered drivers of the origin of indeterminate growth strategy in species taking parental care by offspring brooding in body cavities.

## Graphical abstract

We studied the growth of duck mussels, *Anodonta anatina*, a pan‐European unionid, in 18 Polish lakes representing different trophic conditions. We found two sources of mortality in *A. anatina* populations, pertaining to adverse effects of zebra mussel biofouling and trophy state on mussel survival. In all populations, females featured higher mortality and larger asymptotic size versus males, and consistently for each sex, populations with a greater trophy‐related mortality were characterized in *A. anatina* by a smaller asymptotic size, indicative of a life history response to mortality risk involving early maturation at a smaller body size. Our findings support a theoretical view that adaptive responses to selection involve adjustments in the lifetime resource allocation patterns.

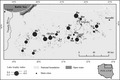

## 1 INTRODUCTION

Unionids are a family of freshwater mussels that commonly occur in freshwater on all continents, except Antarctica (Graf & Cummings, 2007). This group of mussels evolved a complex life strategy that involves larvae called glochidia, which are brooded by females (or hermaphrodites) in gill chambers followed by releasing them into the aquatic environment as fish ectoparasites (Araujo et al., 2005; Barnhart et al., 2008; Hinzmann et al., 2013; Labecka & Domagala, 2018). Upon metamorphosis, parasitic glochidia convert into free‐living filter‐feeders that occupy benthic zones (Zając & Zając, 2011). Another notable aspect of unionids’ life strategy is an incomplete cessation of somatic growth after maturation. Consequently, mature individuals continue alternated allocation to growth and reproduction even up to the end of their lives, which results in so‐called indeterminate growth (Kozlowski, 1996; Kozlowski & Uchmański, 1987; Labecka & Czarnoleski, 2019). The indeterminate growth strategy has evolved in many other species of molluscs, and also in nematodes, annelids, crustaceans, insects, fish, amphibians, reptiles, and plants, but its adaptive value is vague (Stearns, 1992). Life history theory views somatic growth as an investment into the future reproduction capacity or survival with delayed returns—a currently available calorie can be either allocated to growth and therefore be aimed at fitness returns in the future, or to current reproduction, bringing immediate fitness benefits (Czarnoleski & Kozlowski, 1998; Kozlowski, 1996; Stearns, 1992). A range of selection conditions were identified to promote the continuation of somatic growth after maturation, including a strong positive effect of body size on fertility and survivorship as well as seasonal discontinuities reflected in the rates of tissue production and mortality (Kozlowski, 2006; Kozlowski & Teriokhin, 1999), or in the fate of offspring released to the environment (Ejsmond et al., 2010, 2015). For many species, seasonality would be the primary driver of indeterminate growth. However, offspring brooding species such as unionids might be additionally selected for the continuation of growth investments after maturation (Antoł & Czarnoleski, 2018; Labecka & Czarnoleski, 2019). Czarnoleski and Kozlowski (1998) postulated that reproduction via clutches elicits temporal changes in mortality and physiological capacity to produce new tissue, similar in principle to the effects of seasonality. Indeed, Heino and Kaitala (1996) designed an optimal resource allocation model demonstrating that an indeterminate growth strategy evolves even in non‐seasonal environments, given that brooding is costly and leads to a temporary association between the fates of the offspring and their parents. According to Perrin and Sibly (1993), offspring brooders are additionally selected for an indeterminate growth if current offspring production becomes more limited by a brooding space compared to an available physiological capacity.

Addressing the adaptive value of growth strategy, we have studied the growth pattern of the pan‐European long‐term brooding (bradytictic) duck mussel, *Anodonta anatina*, in 18 lakes of north‐eastern Poland. Previously, Müller et al. (2015) indicated that the lakes chosen for this study represent a mosaic of biological and physicochemical parameters, suggesting that the studied populations of *A. anatina* occupy a wide range of selective conditions. Therefore, we expected that the shape of growth curves would be tightly linked to environmental factors with a potential selective value in the way predicted by theoretical evolutionary models. For this purpose, first we integrated available information on lake trophy, mortality in duck mussel populations, infestation of duck mussels by invasive Ponto‐Caspian zebra mussels, *Dreissena polymorpha,* and the prevalence of castrating parasitic trematode larvae. These integrated data helped us identify biotic and abiotic factors that might influence nutritional conditions and mortality rates in the studied populations. We expected that inter‐lake differences in terms of mortality rates in duck mussels would be correlated with the prevalence of parasites and the infestation by zebra mussels. Next, we used our integrated measures of mortality and production conditions to examine their relationships with the growth curves of duck mussels. Following the principles of life history theory (Stearns, 1992), we predicted that (i) populations of higher mortality rates should manifest themselves by an earlier maturation. As a result, this would lead to a negative inter‐population correlation between mortality and an asymptotic body size of duck mussels. It may also be expected that the availability of nutrients stimulates the somatic growth of mussels, leading to a larger asymptotic size of mussels in high‐trophy lakes. Nevertheless, taking into account the results of Czarnoleski et al. (2003, 2005) we predicted that (ii) an increased trophy elevates a degree of infestation by zebra mussels, thereby deteriorating oxygen and nutritional conditions of duck mussels and leading to growth disturbances (Baker & Hornbach, 2000; Mackie, 1991; Sousa et al., 2011). This should constitute a pressure factor for duck mussels’ earlier maturation and, thus, a smaller asymptotic size. For the studied populations of *A. anatina*, Müller et al. (2015) demonstrated that females were more frequently infected with trematode larvae than males. Classic life history models (Stearns, 1992) predict that such a difference in the prevalence of parasites should select females for earlier maturation, resulting in a smaller asymptotic size in females versus males. Yet, with reference to the life history model of Heino and Kaitala (1996), additionally supported by empirical evidence for *Sinanodonta woodiana* mussels (Labecka & Czarnoleski, 2019), we considered that (iii) gill‐brooding might select females for an increased allocation to post‐maturation growth, leading to a larger asymptotic size in females versus males.

## 2 METHODS

### 2.1 Study area and lake choice

**Figure 1:**
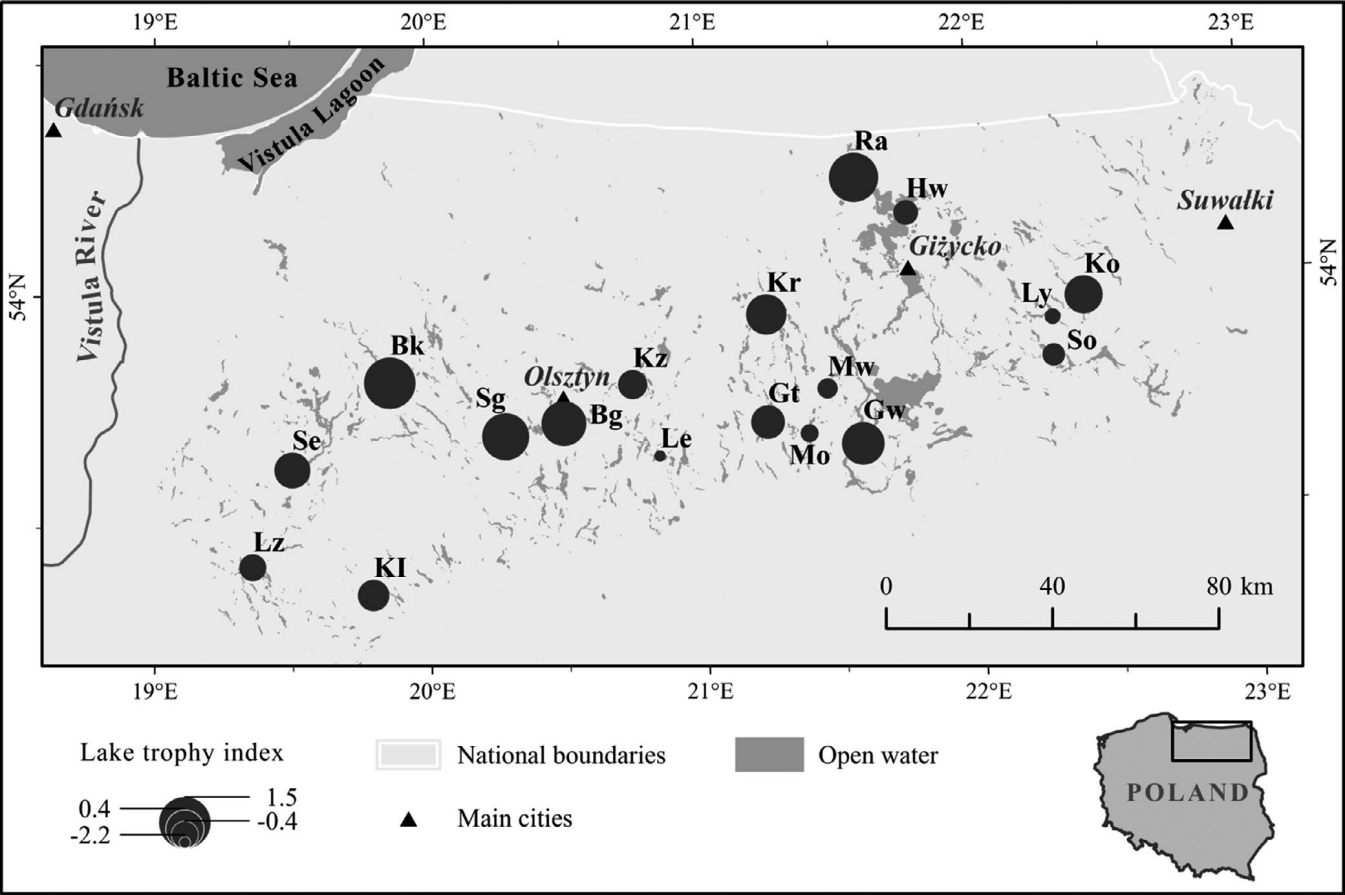
Duck mussels, *Anodonta anatina*, were studied in 18 lakes in north‐eastern Poland, with each lake located in a separate drainage basin: Bartąg (Bg), Bartężek (Bk), Gant (Gt), Guzianka Wielka (Gw), Harsz Wielki (Hw), Kiełpińskie (Kl), Kiersztanowskie (Kr), Kierźlińskie (Kz), Kukowino (Ko), Leleskie (Le), Łąkorz (Lz), Łaśmiady (Ly), Majcz Wielki (Mw), Mokre (Mo), Rydzówka (Ra), Sarąg (Sg), Sunowo (So), and Szymbarskie (Se). The lakes spanned a range of trophic conditions, marked here by a value of the trophy index (for more information about lakes and the trophy index see Tables 1 and 2)

Our study was located in north‐eastern Poland (Figure 1), a vast and thinly populated lake district with around 3,000 lakes, many forests and agriculture fields, with a great deal of the lakes representing moraine‐dammed water bodies created during Pleistocene glacial activity (Pochocka‐Szwarc, 2013). By locating our research in the lake district, we considered that the availability of the fish species that are the hosts for *A. anatina* glochidia would not be a limiting factor here. Females of *A. anatina* utilize a wide range of fish hosts (Lopes‐Lima et al., 2017), which commonly occur in the studied area (long‐term fish monitoring and management are carried out in the studied area by the Inland Fisheries Institute, www.infish.com.pl). The choice of lakes for our study was preceded by the analysis of the available information on the hydrographic properties and trophy‐related parameters in the lakes. We narrowed our focus to the dimictic lakes from different drainage basins, aiming at well‐defined duck mussel populations exposed to a similar mode of temporal fluctuations in the environmental conditions. By considering lakes scattered across a large area (see Figure 1) that were, to a considerable extent, disconnected from one another, we were able to address processes, namely life history responses to environmental conditions in the lakes, which occurred independently in each population.

Our preliminary research identified 30 candidate lakes, which were subsequently examined during the explorative field research undertaken in April 2008. This step aimed at evaluating the presence and abundance of duck mussels in the candidate lakes. Many candidate lakes appeared to be hard to access due to their swampy forested surroundings and wide reed belts on the banks. Therefore, our explorative research helped us identify access points and potential sampling sites in the lakes. Ultimately, we chose to study 18 dimictic lakes (Figure 1 and Table 1), each located in a separate drainage basin (Jańczak, 1999) and characterized by a set of physio‐chemical parameters linked to its trophic status (Müller et al., 2015). Depending on the lake size and mussel abundance, the lakes were represented by 1–3 sampling sites, each characterized by sandy substrate and a confirmed presence of duck mussels.

**Table 1:** The study involved 18 populations of duck mussels (*Anodonta anatina*) inhabiting lakes located in north‐east Poland and representing a variety of drainage basins (see also Figure 1)

	Lake (abbreviation)	Area [km^2^]	Max depth [m]	Mean depth [m]	Volume [m^3^·10^6^]	Drainage basin^a^	Longitude	Latitude	Trophy index
1	Bartąg (Bg)	0.72	15.2	6.4	4.70	LM	53°43.1′	20°29.5′	1.11
2	Bartężek (Bk)	3.78	15.0	5.1	19.61	DE	53°49.4′	19°51.2′	1.49
3	Gant (Gt)	0.75	28.3	9.4	7.12	BMKG	53°42.5′	21°14.2′	0.14
4	Guzianka Wielka (Gw)	0.60	25.5	6.5	3.90	BMN	53°38.7′	21°33.8′	0.87
5	Harsz Wielki (Hw)	2.16	47.0	11.4	24.71	M	54°10.8′	21°46.4′	−0.59
6	Kiełpińskie (Kl)	0.61	11.0	6.1	3.71	WML	53°21.2′	19°47.6′	−0.16
7	Kiersztanowskie (Kr)	1.49	32.5	12.2	18.11	GD	53°56.8′	21°14.5′	0.74
8	Kierźlińskie (Kz)	0.93	44.5	11.7	10.86	P	53°48.2′	20°45.0′	−0.25
9	Kukowino (Ko)	1.28	14.1	5.8	7.42	EŁP	53°57.6′	22°24.5′	0.42
10	Leleskie (Le)	4.24	49.5	12.2	51.80	PKK	53°38.5′	20°50.0′	−2.18
11	Łąkorz (Lz)	1.62	30.3	11.6	18.74	S	53°25.0′	19°21.5′	−0.34
12	Łaśmiady (Ly)	8.82	43.7	9.5	84.61	ELLU	53°55.5′	22°17.4′	−1.35
13	Majcz Wielki (Mw)	1.64	16.4	6.0	9.86	TRJ	53°46.9′	21°27.4′	−0.91
14	Mokre (Mo)	8.41	51.0	12.7	107.33	BMKM	53°41.3′	21°23.3′	−0.93
15	Rydzówka (Ra)	4.90	16.7	6.2	30.94	SR	54°14.1′	21°34.9′	1.22
16	Sarąg (Sg)	1.83	16.5	6.9	12.57	PU	53°41.6′	20°16.8′	1.18
17	Sunowo (So)	1.76	20.6	9.3	15.46	ES	53°50.0′	22°17.1′	−0.61
18	Szymbarskie (Se)	1.65	25.1	6.1	10.07	OU	53°37.8′	19°30.4′	0.15

Note

The characteristics of the lakes were obtained from Jańczak (1999). The trophy index was obtained from Müller et al. (2015); see Table 2 for parameters contributing to the trophy index.

^a^ BMKG—Bełdany Lake, Mikołajskie Lake, Krutynia River, Gant Lake; BMKM—Bełdany Lake, Mikołajskie Lake, Krutynia River, Mokre Lake; BMN—Bełdany Lake, Mikołajskie Lake, Nidzkie Lake; DE—Drwęckie Lake, Elbląg Canal; ELLU—Ełk (a tributary from Sawinda Lake), Łaźna Struga River, Łaśmiady Lake, Ułówki Lake; EŁP—Ełk, Łaźnia Struga River, Połomska Młynówka River; ES—Ełk; GD—Guber River, Dajna River; LM—Łyna (middle course); M—Mamry Lake; OU—Osa River (upper course); P—Pisa River; PKK—Pisa River, Kiermas River, Kalwa River; PU—Pasłęka River (upper course); S—Skarlanka River; SR—Świnia River, Rawda River; TRJ—Tałty Lake, Ryńskie Lake, a tributary from Jorzec Lake; WML—Wel River (middle and lower course).

### 2.2 Mussel sampling and laboratory procedures

All the main procedures used in this study were earlier published by Müller et al. (2015). In September 2008 and 2009, samples of duck mussels were collected, following the recommendations of Strayer and Smith (2003) for mussel field studies. Note that the timing of the mussel collection corresponded to the period of glochidia brooding, which allowed aspects of female fecundity to be studied elsewhere (Müller et al., 2015). Looking for sampling sites, it was considered that duck mussels in lentic ecosystems prefer sandy substrate and depths not exceeding 5 m (Piechocki & Dyduch‐Falniowska, 1993). Within each sampling site, duck mussels were collected by wading and SCUBA diving with the assistance of a small boat, along the transect line stretching the sandy bottom from the shore to the depth of 5 m. Given the logistic limitations, the aim was to obtain around 200 mussels per lake, which ensured the collecting of representatives of both sexes in a wide range of age classes. The actual sample size differed between lakes (176–201), which reflected the mussel availability within the sampling sites and the sampling procedures (to maintain randomness, collecting or discarding individual mussels was avoided after the mass‐sampling). Immediately after collection, the mussels were transported to the field station of the Polish Academy of Sciences in Mikołajki (within the study area), where the laboratory procedures were performed.

Zebra mussels attached to duck mussels were detached and weighed to the nearest 0.01 g on an electronic balance (Kern PCB, Kern & Sohn GmbH), and this information was used to calculate an infestation index (see below). For each duck mussel, adductor muscles were cut across to obtain internal organs for the evaluation of mussel sex and parasitic infections, and shells for the evaluation of shell size and mussel age. Microscopic and histological methods were used to determine mussel sex as well as the infection with digenean trematodes, which was evaluated for the gonads and hepatopancreas of each mussel (results already published by Müller et al., 2015). Shell dimensions were measured to the nearest 0.01 mm with a vernier calliper. The cube root of the product of the shell length, width, and height was used as the final measure of the body size of the mussels (mm). Following Haukioja and Hakala (1978), the annual rings deposited on shells were used to assess the age of the mussels (years). This technique was successfully used in previous works on mussels, including the authors’ research (Czarnoleski et al., 2003, 2005; Labecka & Czarnoleski, 2019; Müller et al., 2015), though note that it can underestimate the age of the oldest mussels that slow down growth (Haag & Commens‐Carson, 2008; Neves & Moyer, 1988). Our study did not aim at absolute values of mussel longevity, so any potential underestimations of these values (if any) would affect all our population datasets, and thus not change the nature of cross‐population relationships that were studied. Data on the body size and age of the mussels were further used to plot growth curves for each population and sex (see below).

In total, 3,535 duck mussels were collected, including 2,111 females, 1,280 males, and 47 hermaphrodites. In the case of 97 individuals with totally spawned gonads or gonads completely damaged by parasites, sex identification was not instituted. The age of mussels was estimated to range from 1.5 to 13.5 years. All further analyses were performed only on individual mussels classified as either males or females. Statistica 13.3 software (TIBCO) was used in all analyses.

### 2.3 Quantifying selective conditions in lakes

It was generally considered that life history strategies are under the selective pressure of agents related to food and mortality conditions (Stearns, 1992). Therefore, to quantify the strength of these agents in the studied populations, the published information on lake trophy and trematode prevalence calculated for the same populations by Müller et al. (2015) was combined with our assessments of the rates of duck mussels’ mortality and infestation by zebra mussels. In contrast to the trophy index that characterized all the mussels in a lake, the indices of mortality, zebra mussel overgrowth, and trematode infection were calculated for every lake separately for *A. anatina* males and females. Ultimately, each lake (duck mussel population) was characterized by a single value of the trophy index (Table 1), and by two sex‐specific values of each of the three indices. Finally, a principal component analysis (PCA) was made on this set of data, which integrated the information on trophy, mortality, overgrowth, and infections. Prior to PCA, the indices of overgrowth and infection were transformed by natural logarithms to attain normality. Scores of the extracted principal components were used in further analyses to test our hypotheses about growth curves (see below).

Müller et al. (2015) characterized the trophic conditions in lakes by fifteen physio‐chemical parameters (Table 2) that were obtained by the Regional Inspectorate of Environmental Protection (Olsztyn, Poland) in the years 2003–2007 as a part of the annual water quality monitoring program (ISO/IEC 17025 standards). The parameters were then analyzed with a factor analysis to acquire an integrated measure of all parameters (a factor that appeared to explain 49% of the variances in parameter values), which was used here as an index of lake trophy. Generally, high values of the trophy index reflect an elevated eutrophication, characterized by reduced oxygen conditions, an increased supply of phosphorous and nitrogen, increased chlorophyll content in the water column, and decreased water visibility (Table 2).

**Table 2:** Results of factor analysis (biquartimax normalized rotation method) of 15 measures of physicochemical conditions in 18 lakes studied here (modified from Table 1 in Müller et al., 2015)

Measure	Unit	Loading
Mean oxygen saturation of hypolimnion _ln_	%	−0.68
Biochemical oxygen demand _ln_ (summer, surface)	mgO_2_/dm^3^	0.51
Biochemical oxygen demand _ln_ (summer, bottom)	mgO_2_/dm^3^	0.70
Phosphates _ln_ (summer, bottom)	mgP/dm^3^	0.92
Total phosphorus _ln_ (summer, bottom)	mgP/dm^3^	0.93
Total phosphorus _ln_ (summer, surface)	mgP/dm^3^	0.76
Ammonium nitrogen _ln_ (summer, bottom)	mgN/dm^3^	0.82
Total nitrogen _ln_ (spring/summer, surface average)	mgN/dm^3^	0.49
Chlorophyll _ln_ (spring/summer, surface average)	mg/m^3^	0.74
Secchi disk _ln_ (spring, summer)	m	−0.80
Maximal depth _ln_	m	−0.61
Water quality index	1.33–3.29	0.87
Ca^2+^ (spring, surface)	mgC/dm^3^	−0.27
pH	−ln_10_(H_3_O^+^)	0.54
Temperature (summer, at 3‐m depth)	°C	−0.49

Note

The first extracted factor shown here explained 49% of the variance in the data, and it was used as a measure of the trophy state of the lakes studied here. Loading the values indicates the contribution of each measure to the trophy state (the extracted factor). High values of this measure of the trophy state reflect the increased eutrophication, generally characterized by decreased oxygen conditions, enhanced supply of phosphorous and nitrogen, increased chlorophyll content in the water column and diminished water visibility.

Unionidae mussels are intermediate hosts in the life cycles of several families of digenean trematodes, with duck mussels being commonly utilized as the first intermediate host by *Rhipidocotyle campanula* (Bucephalidae) (Gibson et al., 1992; Taskinen et al., 1991). To complete its life cycle, *R*. *campanula* further transmits through the next intermediate host, the common roach (*Rutilus rutilus*), later entering the definite host, either the perch (*Perca fluviatilis*) or the zander (*Stizostedion lucioperca*) (Gibson et al., 1992; Taskinen et al., 1991). For the purposes of this study, results already published were used on the occurrence of trematode parasites in the studied populations (Müller et al., 2015). Note that cercariae morphology was used to determine the trematode species (Baturo, 1977; Orecchia et al., 1975; Richardson, 1990; Taskinen et al., 1991). Overall, Müller et al. (2015) demonstrated a common occurrence of *R. campanula* among duck mussels that were studied here, with less abundant infections by *Phyllodistomum* sp. The incidence of infection varied among the studied lakes, ranging from 0.5% in Lake Rydzówka to 27.0% in Lake Gant. Interestingly, parasitic trematode larvae were located in the gonads as well as in the hepatopancreas of duck mussels, but the majority of infections occurred in the gonads. Also note that the between‐lake comparisons of Müller et al. (2015) revealed that a high prevalence of trematodes in a population was associated with a low frequency of brooding and a low number of glochidia incubated by brooding females. Müller et al. (2015) estimated the prevalence of trematode larvae in the studied populations in males and females, which was used here as an index of trematode infection. This index represents the percentage of individuals infected by trematodes, estimated by a statistical model at a standard (grand mean) age of duck mussels, separately for each population and sex. Therefore, the infection index accounts for any differences in the age structure or sex ratio among these mussel population samples, which helped characterize inter‐population differences in the pressure of parasites treated as a selective agent of duck mussels. Additionally, the calculations of Müller et al. (2015) involved information on the mean mass of overgrowing zebra mussels per individual duck mussel in the population; however, this effect turned out to be unrelated to the infection status of mussels.

The mortality rate in duck mussels was quantified with methods developed by Czarnoleski et al. (2003), Czarnoleski et al. (2005). A simple 1/*T*_max_ statistic was implemented to estimate the mean mortality rate calculated separately for female and male mussels in each population (hereafter the mortality index), where *T*_max_ denotes an average age of the oldest mussel in a population sample. As the data from different populations included different sample sizes and the number of female and male mussels varied across populations, a randomization procedure was employed in the calculations of *T*_max_. From each sample of female or male mussels derived from a population data set, a subsample of 10 mussels was randomly taken and the age of the oldest individual was recorded. This procedure was repeated 100 times with replacements, and the values of the age of the oldest individuals from the 100 subsamples (per sex and population) were averaged for calculating *T*_max_. Finally, for each population two sex‐specific mortality indices were obtained, one of which was determined for female and the second for male mussels.

Assessing the pressure exerted on duck mussels by the overgrowing zebra mussels, the potential links between the mass of attached zebra mussels and the shell size of duck mussels were considered. To illustrate this effect, linear regressions were fitted to log‐transformed data on the mass of attached zebra mussels and shell volume of the overgrown duck mussels. The shell volume was calculated from the product of the shell length, width, and height. The regression analysis was carried out separately for each population and sex. It included only those mussels overgrown by zebra mussels. These regressions were used to calculate the mean mass of zebra mussels overgrowing a duck mussel at its standard size (grand mean shell volume among all studied mussels) for each lake. The predicted mass of overgrowing zebra mussels was further used as the index of overgrowth intensity, determined for *A. anatina* females and males from each population.

### 2.4 Explaining variation of growth curves

A two‐parameter Bertalanffy's formula $L\left(t\right)=L_{\max}\left(1‐e^{‐kt}\right)$ was used to find a mathematical representation of growth curve trajectories of female and male mussels in each population (Bertalanffy, 1957; Czarnoleski et al., 2003, 2005; Labecka & Czarnoleski, 2019). The parameter *L*(*t*) denotes body size in linear units at age *t*, *L*_max_ is an asymptotic size, and *k* is the growth rate coefficient indicating how rapidly *L*_max_ is being approached over time. A nonlinear regression model with a least square method using Simplex procedure was chosen to fit the Bertalanffy's formula to the data on age and body size.

Focusing on the variance of Bertalanffy's growth curves, a general linear model (GLM) was used to explore inter‐population and between‐sex differences in the shape of a growth curve. The model included sex as a fixed factor and population as a random factor, and either the asymptotic length *L*_max_ or the growth rate coefficient *k* as dependent variables. Prior to the analysis, data on *L*_max_ and *k* were transformed by natural logarithms to attain normality. A similar GLM was obtained to explore inter‐population and between‐sex differences in the mortality index.

To test our hypotheses (i–iii), a series of multiple regression analyses were made with the asymptotic length *L*_max_ and the growth rate *k* as dependent variables (log‐transformed), and the measures of selective conditions in populations as numerical predictors. Note here that the predictors were estimated with the PCA (see above) and were expressed by scores of extracted principal components. Separate analyses for female and male mussels were completed.

## 3 RESULTS

### 3.1 Quantifying selective conditions in lakes

Principal component analysis extracted two principal components from the input data, which overall accounted for 66% of variance in the input data (Table 3). Interestingly, mortality rate considerably contributed to both principal components. This indicates two distinct sources of mortality, each independent of the other. The first principal component (hereafter PC 1) was correlated negatively with infection indices but positively with overgrowth and mortality indices in duck mussel populations. Therefore, a high score of PC 1 highlights a considerable overgrowth intensity and a high mortality combined with a low prevalence of parasites. The second principal component (hereafter PC 2) was mainly determined by positive effects of parameters describing trophic status and mortality rates. Therefore, a high score of PC 2 indicates an increased trophy combined with a high mortality.

**Table 3:** Principal Component Analysis (PCA) of data on lake trophy, mortality, trematode infections, and zebra mussel overgrowth in 18 populations of *Anodonta anatina* (data on infection level and overgrowth by *Dreissena polymorpha* were log transformed to attain normality) produced two components (PC 1 and PC 2)

Parameters	PC 1	PC 2
Mortality rate (females)	0.54	0.70
Mortality rate (males)	0.48	0.84
Prevalence index (females)	−0.71	0.12
Prevalence index (males)	−0.66	0.27
Overgrowth index (females)	0.82	−0.40
Overgrowth index (males)	0.81	−0.16
Trophy index	−0.10	0.55
Explained variance	40%	26%

Note

Loading values indicate a contribution of each parameter to the PCs. Note that a high value of PC 1 scores indicates that *Anodonta anatina* in a given population experienced high mortality rates combined with a low level of parasitic infection and a high level of overgrowth by zebra mussels. A high value of PC 2 scores indicates high mortality rates combined with high trophy index values.

### 3.2 Explaining variation of growth curves

**Figure 2:**
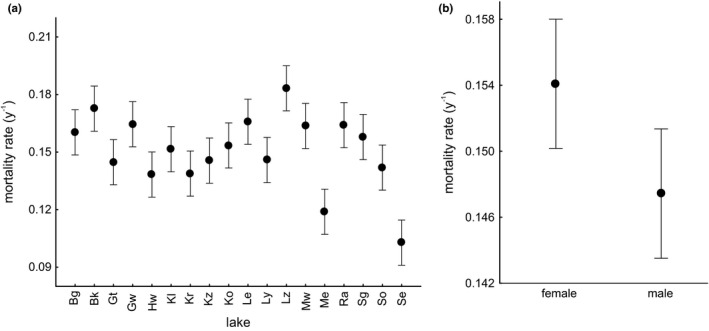
Mortality index of *Anodonta anatina* differed among lakes (a) and between sexes, with females experiencing higher mortality rates than males (b). The mean values (±CI) were estimated with statistical models outlined in Table 4. The lakes are listed in Figure 1 and Table 1

**Figure 3:**
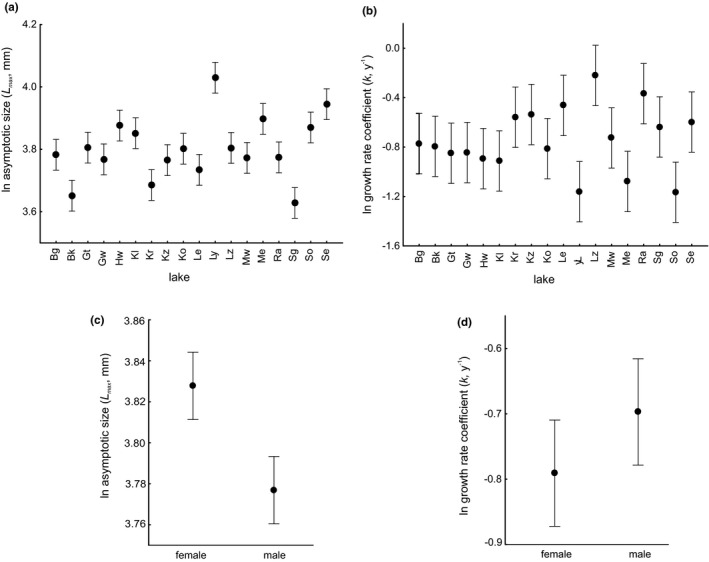
Growth curves of *Anodonta anatina* differed among lakes (a, b) and between sexes (c, d). The shape of the growth curve was described by Bertalanffy's formula, with the asymptotic size *L*_max_ and growth rate coefficient *k*. Mean values (±CI) were estimated with statistical models outlined in Table 4. The lakes are listed in Figure 1 and Table 1

Results of GLM (Table 4) demonstrated that the values of mortality indices substantially differed among populations (*p* < 0.001) and females experienced higher mortality rates than males (*p* = 0.02) (Figure 2a,b). Results of another GLM (Table 4) yielded a significant variation in the shape of growth curves among populations (*p* < 0.001) (Figure 3a,b), and sex‐specific differences in growth curves with female mussels attaining a larger asymptotic size than males (*p* < 0.001) (Figure 3c). Bertalanffy's growth rate coefficient *k* tended to be smaller in females compared to males (*p* = 0.10) (Figure 3d).

**Table 4:** Results of General Linear Models comparing mortality index and growth curve parameters of the duck mussel *Anodonta anatina* between sexes and among 18 lake populations

Dependent variable	Predictor	Effect	*df*	*F*	*p*
Mortality index	Population	Random	17	11.76	<0.001
	Sex	Fixed	1	6.41	0.02
	Error		17		
Asymptotic size *L*_max_	Population	Random	17	18.24	<0.001
	Sex	Fixed	1	21.35	<0.001
	Error		17		
Growth rate coefficient *k*	Population	Random	17	5.04	<0.001
	Sex	Fixed	1	2.96	0.10
	Error		17		

Note

Parameters *L*_max_ and *k* denote an asymptotic size and a growth rate coefficient of Bertalanffy's growth curves, respectively.

Our multiple regression analysis (Table 5) demonstrated that the scores of PC 1 correlated neither with asymptotic size *L*_max_ nor with Bertalanffy's growth rate coefficient *k*. It means that the exposure of duck mussels to digenean trematodes or infestation by zebra mussels did not change their growth trajectory. However, we found a negative correlation between the scores of PC 2 and *L*_max_. Such a pattern was consistently observed in females and males. In males, there was also a tendency for PC2 to be positively correlated with *k*. Overall, these results indicated that mussels attained a smaller asymptotic size in populations with higher mortality combined with high‐trophic levels.

**Table 5:** Results of four Multiple Regression analyses (partial correlation coefficients *R* with *p*‐values) of growth curve trajectories in 18 populations of *Anodonta anatina* in relation to environmental conditions (indices PC 1 and PC 2)

	Dependent variable	Explanatory variables			
		PC 1		PC 2	
		*R*	*p*	*R*	*P*
Females	*L*_max_	−0.16	0.48	−0.52	0.03
	*k*	−0.01	0.97	0.27	0.29
Males	*L*_max_	−0.30	0.12	−0.66	<0.01
	*k*	0.27	0.25	0.41	0.09

Note

Parameters *L*_max_ and *k* denote an asymptotic size and a growth rate coefficient of Bertalanffy's growth curves, respectively. The values of PC 1 and PC 2 represent scores of two principal components derived from the PCA analysis of biotic and abiotic conditions in the studied lakes (Table 3).

## 4 DISCUSSION

The Unionidae family comprises *ca*. 700 species (Lopes‐Lima et al., 2020), including many endangered species (Lopes‐Lima et al., 2017; Lydeard et al., 2004; Zieritz et al., 2018), species characterized by unique reproductive biology (Barnhart et al., 2008; Labecka & Domagala, 2018, 2019; Vicentini, 2005), or species regarded as pivotal elements for ecosystem functioning or ecosystem services (Spooner et al., 2013; Strayer, 2014; Vaughn, 2018; Vaughn & Hakenkamp, 2001). Despite a vast common interest in unionids, we know surprisingly little of how this group of molluscs responds to environmental heterogeneity of habitats. Our comparative data for 18 Central European lakes help us identify factors that may impose selective pressure on duck mussels, resulting in adaptive responses of their life history strategy.

Having analyzed data on lake trophy with reference to information on the characteristics of duck mussel populations (parasitism, biofouling, mortality), we found two distinct patterns expressed by our principal components (PC 1 and PC 2). The nature of these two patterns indicates that the mortality rate of duck mussels considerably differed among lakes and this variance had two independent sources, one related to the effects of zebra mussel biofouling and the other related to the effects of lake trophy. The structure of the first pattern (PC 1) suggests that zebra mussel biofouling was responsible for a rise in mortality in duck mussel populations. The spread of Ponto‐Caspian zebra mussels across freshwaters in North America at the end of the 20th century is often regarded as a serious extinction threat to the native Unionidae fauna (Schloesser et al., 1996; for comparison see also Strayer & Malcom, 2007), with the long‐term brooding species of unionids being considered the most vulnerable group (Haag et al., 1993). The species composition of North American unionids is different from that in European unionids, and we may generally expect that unionids respond (e.g., with shell growth or life history) to zebra mussel biofouling in a species‐specific manner (Burlakova et al., 2000; Dzierżyńska‐Białończyk et al., 2018; Haag et al., 1993). European unionids like *A. anatina* might be less susceptible to zebra mussel biofouling than their North American counterparts because of their much longer sympatry with zebra mussels (Burlakova et al., 2000; Sousa et al., 2011; Welter‐Schultes, 2012). In fact, we have not detected any effects of zebra mussel biofouling on the growth pattern of the studied duck mussels (Table 4), which might suggest that, against our hypothesis (ii), zebra mussels do not considerably affect the resource allocation strategy of duck mussels.

The analysis of our PC 1 suggests that populations of duck mussels characterized by intense zebra mussel biofouling were simultaneously less threatened by digenean trematode infections. On one hand, biofouling might worsen physiological conditions by reducing glycogen content and/or protein production, among other things. This can lead to decreased immunocompetence and increased vulnerability to infections (Baker & Hornbach, 2000, 2008; Haag et al., 1993; Sousa et al., 2011). On the other hand, however, the risk of contracting planktonic parasites by filter‐feeding mussels seems to be tightly linked to the total amount of water processed by the host, and perhaps, it explains why some studies of unionids reported that trematode infections were increased along with mussel age (Müller et al., 2015; Taskinen & Valtonen, 1995). This mechanism can also account for our findings because zebra mussel biofouling restricts shell movements and filtration activity (Czarnoleski et al., 2003; Müller et al., 2015; Schloesser et al., 1996). Hence, it should reduce water processing by gills, thereby leading to a negative association between overgrowth intensity and digenean trematode infections in duck mussels. Alternatively, although not exclusively at the same time, zebra mussels could more directly reduce the exposure of overgrown duck mussels to trematodes by filtering out, and perhaps even digesting, planktonic stages of the parasites. It is telling that zebra mussels seem to be resistant to *R. campanula* and *Phyllodistomum* sp. trematodes (Marszewska & Cichy, 2015), while these parasites commonly occur in the *A*. *anatina* populations studied here (Müller et al., 2015). Certainly, future studies should rigorously test which of the two mechanisms discussed here might be the real force shaping the prevalence of trematode infections in duck mussel populations.

Given the structure of our PC 1 pattern, we did not find evidence on any relationship between the intensity of zebra mussel biofouling and lake trophy. In contrast, Czarnoleski et al. (2003, 2005) presented evidence that a reduced availability of hard substratum in eutrophic lakes intensifies the settlement of planktonic stages of zebra mussels on older conspecifics, resulting in an increase of self‐overgrowth in nutrient‐rich lakes. Although lake trophy did not appear to affect the biofouling of duck mussels by zebra mussels in our study, the structure of our second environmental pattern (PC 2) indicates that more eutrophic lakes were characterized by the higher mortality of resident duck mussels, which accords with our hypothesis (ii). Earlier studies identified different mechanisms linking high nutrient loading, deteriorated living conditions and increased mortality of freshwater mussels, including changes in intraspecific and interspecific competition for food and other resources (Baker & Hornbach, 2000, 2008; Czarnoleski et al., 2003; Hörmann & Maier, 2006; Strayer, 1999), food quality (Basen et al., 2011) and changes in juvenile recruitment (Strayer, 1999; Strayer & Malcolm, 2012), not to mention the origin of anoxic zones with their adverse direct effects on living things (Galbraith et al., 2010).

Focusing on growth patterns, we found notable differences among lakes in the shape of individual growth curves of duck mussels. To maximize an expected lifetime reproductive success, organisms should adjust their schedule of resource allocation and the resulting growth pattern to the selective pressure of the environment, mainly to factors that affect the capacity to produce new tissues and survive (Heino & Kaitala, 1996; Kozlowski, 1996; Perrin & Sibly, 1993; Stearns, 1992). In agreement with this view and our hypothesis (i), we found that duck mussels from low‐mortality/low‐trophy populations attained larger asymptotic size (both females and males) than mussels from the high‐mortality/high‐trophy populations. This pattern suggests that when faced with high mortality associated with water trophy, duck mussels shorten their juvenile period, maturing earlier and at a smaller body size, which ultimately leads to the attainment of smaller asymptotic sizes. It should be stressed here that contrary to (ii) hypothesis, this relationship among lake trophy, mortality, and duck mussels growth pattern was probably not mediated by the effect of lake trophy on the intensity of zebra mussel biofouling (see an earlier part of Discussion). Interestingly, such mediation was earlier postulated as the cause of cross‐population and temporal shifts of growth in European zebra mussels, with intense biofouling by conspecifics resulting in the increased mortality and smaller asymptotic size of zebra mussels (Czarnoleski et al., 2003, 2005). Our results for growth curves also do not support the idea that digenean trematodes imposed changes in the allocation strategy of duck mussels. Similarly, Jokela et al. (1993) reported that the growth pattern of *A. anatina* (*A*. *piscinalis*) remained unchanged regardless of the presence of *Rhipidocotyle fennica* trematode. Yet, other works revealed that digenean trematodes can either negatively (Taskinen, 1998) or positively (Taskinen & Valtonen, 1995) affect the mussel growth. Notably, Taskinen and Valtonen (1995) questioned the nature of causal relationships between the presence of parasites and an enhanced growth, envisioning that parasites can either stimulate mussel growth or more easily infect fast‐growing mussel hosts, thus more intensely filtering them.

In long‐term brooders, such as duck mussels in Poland that carry broods from late summer till the following spring (Domagala et al., 2004; Piechocki, 1969), reproduction incurs substantial costs to females, for example, by increasing female mortality and/or impairing food intake. Indeed, Haag et al. (1993) reported a higher mortality in females compared to males of *Lampsilis radiata* (Unionidae) and our results for duck mussels demonstrated a higher mortality in females versus males. In the *Pyganodon cataracta* unionid, brooding females had lower filtration rates than non‐brooding females (Tankersley, 1996). Brooding also temporarily links the fate of a female with the fate of its offspring, and by developing larger shells, unionid females can provide a larger space for their broods in gill chambers (Kotrla & James, 1987; Labecka & Czarnoleski, 2019). This should result in an increased reproductive capacity of larger females. Heino and Kaitala (1996) used resource allocation modeling to demonstrate that all these effects of brooding select unionid females for the intense somatic growth following maturation. Indeed, we found sex differences in the growth pattern of duck mussels, and following our (iii) hypothesis, the brooding sex (females) attained a larger asymptotic size than the non‐brooding sex (males). This indicates that females presented a higher tendency for the post‐maturation growth than males. Published information on sex‐specific growth of unionids creates an inconsistent picture, with some papers reporting that females reach larger sizes than males (Labecka & Czarnoleski, 2019; Zieritz & Aldridge, 2011) and others indicating no apparent size differences among sexes (Dudgeon & Morton, 1983; Reis & Araujo, 2016).

## 5 CONCLUSION

Using comparative data from different lakes, we searched for factors that might shape the growth strategy in duck mussels. We found large cross‐population differences in the growth pattern of mussels, largely attributable to adaptive shifts of resource allocation to mortality conditions mediated by the trophic state of lakes. We also found that female duck mussels grew toward larger asymptotic sizes and suffered higher mortality than males, indicating that selection factors imposed different pressure on sexes, with different life history responses in each sex. The sex differences in growth pattern support an idea that parental care (here brooding of females) might favor a continuous allocation of resources to growth, resulting in an indeterminate growth pattern. To sum up, our findings strongly suggest that adaptive shifts in resource allocation should be taken into account in order to better understand the origin of cross‐population and sex difference organisms’ growth.

## CONFLICT OF INTEREST

The authors declare no conflicting interests.

## AUTHOR CONTRIBUTIONS

**Tomasz Muller:** Conceptualization (lead); Data curation (equal); Formal analysis (equal); Funding acquisition (lead); Investigation (equal); Methodology (equal); Supervision (lead); Writing‐original draft (lead); Writing‐review & editing (lead). **Anna Maria Labecka:** Investigation (lead); Methodology (lead); Resources (equal); Supervision (equal); Validation (equal); Writing‐review & editing (equal). **Katarzyna Zajac:** Conceptualization (equal); Funding acquisition (supporting); Investigation (supporting); Methodology (supporting); Validation (supporting); Writing‐review & editing (supporting). **Marcin Czarnoleski:** Conceptualization (lead); Formal analysis (lead); Funding acquisition (equal); Investigation (equal); Methodology (equal); Resources (equal); Validation (equal); Writing‐original draft (equal); Writing‐review & editing (lead).

## Data Availability

Quantitative data on selective factors in lakes and data on mussel growth curve parameters available via DRYAD (https://doi.org/10.5061/dryad.nk98sf7sg).
